# A systematic review and meta-analysis of nab-paclitaxel mono-chemotherapy for metastatic breast cancer

**DOI:** 10.1186/s12885-021-08441-z

**Published:** 2021-07-18

**Authors:** Haili Lu, Siluo Zha, Wei Zhang, Qiang Wang, Daozhen Jiang, Xinyun Xu, Xiangmin Zheng, Ming Qiu, Chengxiang Shan

**Affiliations:** Third Division of Department of General Surgery of Changzheng Hospital affiliated with Naval Military Medical University, Fengyang Road, Shanghai, 200003 China

**Keywords:** Nab-paclitaxel, Metastatic breast cancer, Safety, Efficacy, Meta-analysis

## Abstract

**Background:**

Although various clinical trials and real-life studies have tried to explore the value of nab-paclitaxel mono-chemotherapy for metastatic breast cancer (MBC), the safety and efficacy of nab-paclitaxel remain unclear which need to be systematically evaluated.

**Methods:**

Electronic searches for prospective clinical trials evaluating nab-paclitaxel monotherapy for MBC were performed. Requisite data were extracted, integrated and analysed from the included studies according to the different study designs using systematic review and meta-analysis. Meta-regression and subgroup analysis were further performed to explore the potential risk factors affecting each individual outcome of interest following nab-paclitaxel monotherapy.

**Results:**

Twenty-two studies with 3287 MBC patients were included. A total of 1685 MBC patients received nab-paclitaxel as first-line therapy, 640 patients as further-line therapy, and 962 patients as mixed-line therapy. A total of 1966 MBC patients (60.40%) received nab-paclitaxel weekly, 1190 patients (36.56%) received nab-paclitaxel triweekly and 99 patients (3.04%) received nab-paclitaxel biweekly. The overall incidence rates of all-grade neutropenia, leukopenia, peripheral sensory neuropathy, and fatigue were 52% (95% CI, 38–66%, I^2^ = 98.97%), 58% (95% CI, 43–73%, I^2^ = 97.72%), 58% (95% CI, 48–68%, I^2^ = 97.17%), and 49% (95% CI, 41–56%, I^2^ = 94.39%), respectively. The overall response rate (ORR) was 40% (95% CI, 35–45%, I^2^ = 98.97%), and the clinical benefit rate (CBR) was 66% (95% CI, 59–73%, I^2^ = 98.97%) following nab-paclitaxel monotherapy. The median progression-free survival (PFS) was 7.64 months (95% CI, 6.89–8.40 months, I^2^ = 92.3%), and the median overall survival (OS) was 24.51 months (95% CI, 21.25–27.78 months, I^2^ = 92.7%). Treatment line, human epidermal growth factor receptor-2(Her-2)-negative status and dosage were found to be sources of heterogeneity among the included studies. According to the meta-regression and subgroup analysis, grade 3/4 neutropenia occurred less frequently in Her-2-negative patients than in the entire population (*P* = 0.046). Patients who received first-line nab-paclitaxel monotherapy showed a higher ORR (*P* = 0.006) and longer PFS (*P* = 0.045). Efficacy outcomes were not affected by the administration schedule. However, within the same schedule, patients appeared to have a superior ORR (*P* = 0.044) and longer PFS (*P* = 0.03) with an increasing dosage of nab-paclitaxel administered.

**Conclusions:**

The benefits brought by nab-paclitaxel mono-chemotherapy in the treatment of MBC are considerable while the harm is generally manageable. Further study and validation are needed to figure out the roles which the dosage, schedule and other factors play actually in nab-paclitaxel chemotherapy.

**Supplementary Information:**

The online version contains supplementary material available at 10.1186/s12885-021-08441-z.

## Background

Approximately one-fourth of patients with early localized breast cancer will eventually develop recurrent or metastatic breast cancer (MBC) [33]. Once breast cancer becomes metastatic, it is rarely curable, even though mortality has been decreasing steadily in developed countries over the last decade [10]. Although no randomized evidence comparing therapy with observation in women with MBC exists, it is widely recommended that women with MBC should receive some form of systemic therapy during the course of their disease [20]. Chemotherapy has been the cornerstone in the treatment of MBC for many years, and it is generally accepted that taxanes are among the most active single agents [41].

The clinical approval of taxanes for MBC began with paclitaxel in 1994, continued with docetaxel in 1996 and was further updated with nanoparticle albumin-bound paclitaxel (Abraxane, nab-paclitaxel) in 2005 [9]. Although paclitaxel and docetaxel have proven to be clinically beneficial, their hydrophobic chemical formulations have presented obvious limitations [40]. Nab-paclitaxel was developed to eliminate the solvent-related toxicities typically associated with taxane administration. More importantly, this colloidal suspension was also designed to preferentially deliver paclitaxel to tumours by biologically interacting with albumin receptors that mediated drug transport [28]; in vitro studies have demonstrated a 4.5-fold increase in paclitaxel transport across endothelial cells for nab-paclitaxel compared to conventional taxanes [13].

Since Ibrahim et al. [24] first reported a 48% overall response rate (ORR) for 63 MBC patients in a phase II trial of 300 mg/m^2^ nab-paclitaxel monotherapy triweekly, various clinical trials and real-life studies have tried to explore the safety and activity of nab-paclitaxel in treating MBC. Most recently, in the NABUCCO observational study, Marschner et al. [32] reported that nab-paclitaxel monotherapy could offer a 37.2% ORR, a 68.3% clinical benefit rate (CBR), a median time to progression (TTP) of 5.9 months and a median overall survival (OS) of 15.6 months, with lower (5%) grade 3/4 treatment-related adverse events (TRAEs) in 697 MBC patients. Head-to-head clinical comparisons between nab-paclitaxel and conventional taxanes were not rare. Two pivotal phase II/III clinical trials reported by Gradishar et al. [15, 19]) concurrently demonstrated superior efficacy and safety of nab-paclitaxel compared with paclitaxel (175 mg/m^2^, q3w) or docetaxel (100 mg/m^2^, q3w), with a statistically higher ORR, clinically significant prolongation of progression-free survival (PFS), shorter infusion schedules (30 min) and no premedication. However, contradictory results have also been reported. In a phase II multicentre trial with 197 human epidermal growth factor receptor-2(Her-2)-negative patients with MBC, Tamura et al. [39] reported similar efficacy outcomes in patients treated with weekly nab-paclitaxel (150 mg/m^2^) and triweekly docetaxel (75 mg/m^2^). Nab-paclitaxel did not show superiority in PFS compared with docetaxel. In the CALGB 40502 study, Rugo et al. [37] also failed to demonstrate superiority of nab-paclitaxel given weekly compared with paclitaxel in 542 MBC patients; increased overall toxicity with nab-paclitaxel was observed. The authors suggested that weekly paclitaxel should remain the preferred microtubule inhibitor for the first-line therapy of MBC. One prior meta-analysis, including 4 randomized controlled trials (RCTs) with 1506 MBC patients, demonstrated that nab-paclitaxel-based chemotherapy could be associated with increased sensory neuropathy and equivalent survival compared with conventional taxane-based chemotherapy [27]. Therefore, since the superiority of nab-paclitaxel is still controversial and considering that a proportion of studies on nab-paclitaxel monotherapy have had single-arm, non-randomized phase II trials with rather small sample sizes, the safety and efficacy of nab-paclitaxel need to be thoroughly examined.

Although nab-paclitaxel was initially approved by the US Food and Drug Administration (FDA) with a recommended triweekly 260 mg/m^2^ dosage for MBC, evidence suggests that a weekly nab-paclitaxel regimen could also be feasible for patients with MBC, as weekly paclitaxel administration appears to be the optimal schedule for MBC [28]. In fact, a retrospective study reported by Dent et al. [11] showed inferior ORR (4.7% vs. 14.3%) and CBR (57.1% vs. 76.2%) and shorter median OS (10.8 months vs. 13.6 months) in the triweekly nab-paclitaxel group than in the weekly nab-paclitaxel group. Gradishar et al. [16] also demonstrated better disease control for nab-paclitaxel monotherapy qw 3/4 regimens (100 mg/m^2^ and 150 mg/m^2^) versus a q3w regimen, and nab-paclitaxel at 150 mg/m^2^ qw 3/4 resulted in a 33.8-month OS longer than historically achieved with single-agent taxane therapy in MBC. However, in the NABUCCO study, efficacy superiority with respect to better tumour control and longer survival outcomes was not obtained in the weekly nab-paclitaxel group [32]. Irrespective of the survival outcomes, the weekly nab-paclitaxel regimen seemed to increase paclitaxel-related toxicity. Tamura et al. [39] reported that 150 mg/m^2^ qw 3/4 nab-paclitaxel monotherapy would result in a 78% prevalence of grade 3/4 neutropenia and 22% prevalence of grade 3/4 neuropathy in 98 MBC patients. Decreased quality of life (QOL) due to more TRAEs in MBC patients seemed counteract the goal of MBC treatment [12]. Since no tailored regimens were strongly recommended, variable studies with different nab-paclitaxel dosages and schedules were introduced into clinical practice [31], and these data called into question which regimen of nab-paclitaxel was optimal for MBC.

To date, nab-paclitaxel has been suggested to be quite important as a single agent for the first- or further-line treatment of MBC [26]. With the goal of understanding the safety and efficacy following nab-paclitaxel mono-chemotherapy, we reviewed the clinical evidence with nab-paclitaxel as a single agent in metastatic treatment settings and made efforts to distill a clear conclusion without the limitation of each single study. Furthermore, evidence-based optimal regimens and schedules of nab-paclitaxel for MBC were also explored by meta-regression and subgroup analysis.

## Methods

### Study search strategy

According to the Preferred Reporting Items for Systematic Reviews and Meta-Analyses (PRISMA) guidelines [30], a systematic search was independently performed by 2 investigators (Shan CX and Lu HL) using electronic databases, including PubMed/Medline, ClinicalTrial.gov and the Cochrane Center Register of Controlled Trials, to identify articles published between March 2005 and March 2020 using the following search keywords, “nab-paclitaxel” or “albumin-bound paclitaxel” or “abi-007” or “Abraxane”, and “metastatic breast cancer”. The “related articles” function was used to broaden the search. All abstracts, studies and citations were checked for additional relevant material, when appropriate. In addition, abstracts from annual meetings of the American Society of Clinical Oncology (ASCO), European Society of Medical Oncology Conference (ESMO) and San Antonio Breast Cancer Symposium (SABCS) were retrieved for relevant abstracts identified using similar search terms. No language restrictions were imposed.

### Study selection criteria

Abstracts or full-text articles were initially screened and then selected or rejected by the two reviewers (Shan CX and Lu HL) on the basis of the inclusion and exclusion criteria described below.

Inclusion criteria: (1) Designed prospective trials, including both observational studies and interventional studies. (2) Phase II clinical trials, phase III clinical trials and cohort studies. (3) Single-arm, two-arm or multi-arm trials that contained a nab-paclitaxel monotherapy treatment group were all included. (4) The exact dichotomous and continuous data needed to calculate the standard deviation or standard error should be provided to determine the weight of each study.

The exclusion criteria were as follows: (1) Retrospective observational studies. (2) Total sample size < 10 patients. (3) Studies with the same research subjects published repeatedly by different journals.

### Quality assessment

The methodological quality of RCTs was assessed by the Cochrane Collaboration tool according to six domains: random sequence generation, allocation concealment, blinding of investigators and participants, blinding of outcome assessor, completion of outcome data, and selective reporting. All of the domains are graded as “low risk”, “high risk” or “unclear risk” of bias. If no less than 4 “low risk” domains were identified in a trial with no “high risk” domains, the trial was considered low risk and high quality.

The Newcastle-Ottawa Scale was used to evaluate the quality of cohort studies. Grading criteria included representativeness of the exposed cohort, selection of the nonexposed cohort, ascertainment of exposure, demonstration that the outcome of interest was not present at start of the study, comparability of cohorts on the basis of the design or analysis, assessment of outcome, and adequate follow-up. The maximum score was nine.

For non-randomized trials, quality assessment was performed by the Methodological Index for Non-randomized Studies (MINORS). For trials without a control group, eight criteria were required for evaluation: a clear objective, consecutive participants, collection of the expected result, a terminal point that properly reflected the purpose, objectivity of the endpoint evaluation, adequate follow up, less than 5% loss to follow-up, and sample size estimation. For studies with a control group, four additional criteria were also required: appropriate choice of control group, control patients selected at the same period, comparability of the two groups, and optimal statistical analysis. The criteria were graded from zero to two points according to whether the information was reported rarely, inadequately or in detail. The maximum score was 16 or 24.

### Data extraction

The two reviewers independently extracted details from each eligible study:(1) information and quality of the research: first author, year of publication, study design, treatment line, population, sample capacity; (2) nab-paclitaxel regimen, including the dosage and schedule; (3) assessment data (trials containing multiple groups were initially divided into single nab-paclitaxel mono-chemotherapy group and other groups, and then extracted individually); (4) toxicity profile, including the incidence of all-grade and grade 3/4 neutropenia, leukopenia, peripheral sensory neuropathy and fatigue; (5) disease control rate, including the ORR and CBR; (6) survival endpoints, including median PFS and median OS. Specifically, the assessment data of repeated trials published in different journals at different times were extracted from the latest and most detailed article. Furthermore, if the safety and efficacy outcomes were both assessed by radiologists and investigators independently in certain studies, we retrieved the data provided by the investigator.

### Statistical analysis

All trials referring to nab-paclitaxel monotherapy were first split into groups according to nab-paclitaxel administration dosage and schedule. The outcome measures included the incidence of TRAEs, the disease control rate (ORR, CBR), and median survival time (median PFS, median OS). Data analysis in our study was performed with STATA (version 14.0) software. For the ratio analysis, we performed single-arm meta-analysis and adopted the “metaprop” command set in STATA for data integration. An additional “ftt” command set was applied if the rate was unusual high or low. For the survival outcome analysis, median PFS or median OS, with the associated sampling error, were utilized for data integration. The sampling error was either retrieved through the published data or calculated with the following formula: (upper bound of the 95% confidence interval - the lower bound of the 95% confidence interval (CI))/2*1.96.

First, forest plots were generated. If significant heterogeneity existed between the recruited groups (I^2^ > 50%), we adopted a random-effects model; if not, a fixed-effects model was applied. Then, sensitivity analysis was used to evaluate the stability of the results by removing one or more groups at a time to examine the influence of individual studies on the pooled estimate. Generally, if the estimate of a single group was beyond the lower and upper confidence interval limits in the sensitivity analysis, this group was excluded from the next meta-regression analysis. Publication bias was assessed through the Begg and Egger methods in STATA and further represented using funnel plot analysis if publication bias existed with a *p* value < 0.05.

For the heterogeneity source analysis, meta-regression was performed by our consulting professional statistician using the STATA software “metareg” command, and the threshold for significance was set at *p* < 0.05. If a heterogeneity source existed, subgroup meta-analysis was further performed to demonstrate the difference between individual groups due to the specified heterogeneity. Meta-regression was also used to explore the potential risk factors affecting each individual outcome of interest following nab-paclitaxel monotherapy. In our study, PFS, OS, ORR, CBR and TRAEs were as the outcome variables of interest. Treatment line, Her-2 negative population, nab-paclitaxel dosage, and nab-paclitaxel schedule served as explanatory variables.

## Results

### Identification and characteristics of the included studies

**Fig. 1:**
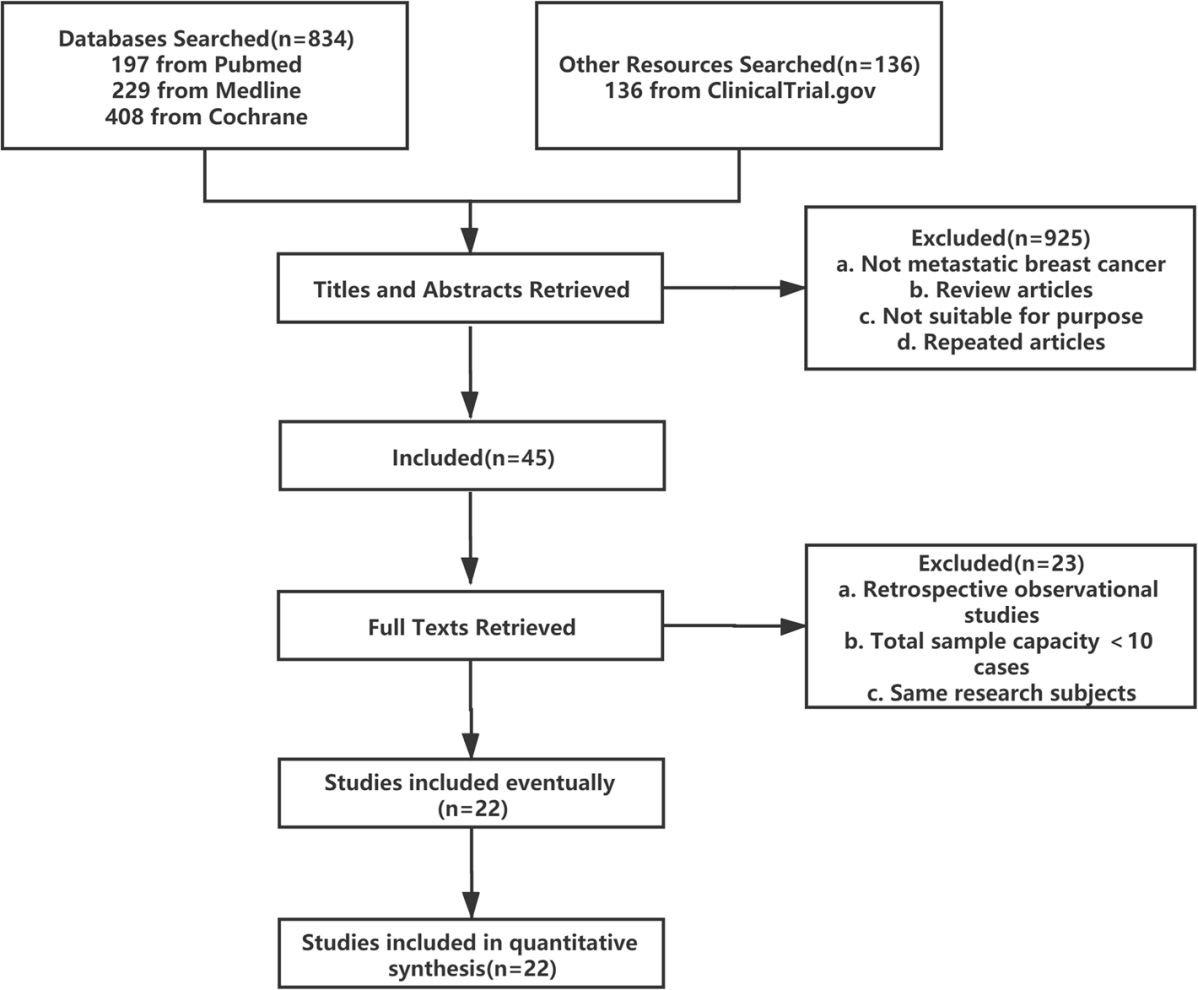
Study retrieval flow diagram

We ultimately identified 22 independent studies published between March 2005 and March 2020 [1, 4–7, 14, 16–19, 21–25, 29, 32, 35, 36, 38, 39, 42]. A flow chart illustrating the selection of studies is shown in Fig. 1. In total, 11 RCTs, 10 non-randomized trials and 1 cohort study were included. The quality of the 11 RCTs was demonstrated to be low risk (Grade A). The single cohort study was also evaluated as high quality (maximum score). Based on the MINORS criteria, five non-randomized trials scored zero points for the sample size estimation criterion [5, 6, 21, 24, 32], one trial scored one point for inadequate information [42], and another trial [5] also scored one point in the follow-up domain for a higher rate (> 5%) of loss to follow-up. The quality assessment of the included studies is presented in Supplement 1. The baseline characteristics of the included studies for MBC are summarized in Table 1. The safety and efficacy profiles of nab-paclitaxel therapy are recorded in detail in Table 2.

**Table 1:** Baseline characteristics of the included studies for MBC

Author + Year	Study Capacity (N)	Her2 Positive (N)	Triple Negative (N)	Median/Mean Age	Therapy Line	Menopausal Status	Setting	ECOG^a^
Ibrahim NK 2005 [24]	63	NM^b^	NM	48.2	Mixed	Both Pre- and Post-	Metastatic	0–1
Gradishar WJ 2005 [19]	454	NM	NM	53.1	Mixed	Both Pre- and Post-	Metastatic	0- ≥ 3
Blum JL 2007 [6]	181	72	28	53	Further	Both Pre- and Post-	Metastatic	0–1
Guan ZZ 2009 [17]	210	NM	NM	50	Further	Both Pre- and Post-	Metastatic	0–1
Mirtsching B 2011 [29]	72	22	NM	63.5	First	NM	Metastatic	0–2
Gradishar WJ 2009/2012 [15, 16]	300	NM	NM	NM	First	Both Pre- and Post-	Metastatic	0–2
Brezden B 2016 [4]	123	NM	NM	NM	First	Both Pre- and Post-	Metastatic	0–2
Ranade AA 2013 [36]	195	NM	NM	NM	Mixed	NM	Metastatic	0–2
Palumbo R 2015 [35]	52	0	16	53	Further	Both Pre- and Post-	Metastatic	0–2
Fabi A 2015 [14]	42	5	8	48	Further	NM	Metastatic	0–2
Hurria A 2015 [21]	39	NM	NM	60	Mixed	NM	Metastatic	0–1
Andres FT 2015 [1]	64	0	64	51	Mixed	NM	Metastatic	0–2
Jain MM 2016 [25]	180	NM	NM	NM	Mixed	Both Pre- and Post-	Metastatic	0–2
Yamamoto S 2017 [42]	35	13	8	59	Mixed	Both Pre- and Post-	Metastatic	0–2
Tamura K 2017 [39]	197	0	36	NM	First	NM	Metastatic	0–1
Bernardo A 2017 [5]	234	0	51	58	Further	NM	Metastatic	0- ≥ 2
Gennari A 2018 [18]	255	0	NM	58	First	NM	Metastatic	0–1
Marschner N 2018 [32]	697	96	96	62.3	Mixed	NM	Metastatic	0- ≥ 2
Hurria A 2019 [23]	40	NM	10	73	Mixed	NM	Metastatic	NM
Ciruelos E 2019 [7]	60	0	NM	NM	First	NM	Metastatic	0–1
Hara F 2019 [22]	141	0	141	NM	Mixed	NM	Metastatic	0–1
Schmid P 2020 [38]	1271	0	1271	NM	First	NM	Metastatic	0–2

^a^*ECOG* Eastern Cooperative Oncology Group

^b^*NM* Not Mentioned

**Table 2:** Safety and efficacy profiles of the included studies for MBC

Author+Year	Study Design	Sample Size (N)	Treatment Line	Nab-paclitaxel Monotherapy Regimen	Treatment Related Adverse Events (TRAEs)								Efficacy Outcomes			
					Neutropenia (%)		Leukopenia (%)		Neuropathy (%)		Fatigue (%)		ORR (%)	CBR (%)	Median PFS (Months)	Median OS (Months)
					All grades	3/4 grade	All grades	3/4 grade	All grades	3/4 grade	All grades	3/4 grade				
Ibrahim NK 2005 [24]	Phase II Multicenter	63	Mixed	300 mg/m^2^ q3w	9	51	91	24	64	11	40	13	48			14.8
		39	First	300 mg/m^2^ q3w									64			
		24	Further	300 mg/m^2^ q3w									21			
Gradishar WJ 2005 [19]	Phase III RCT Multicenter	229	Mixed	260 mg/m^2^ q3w		30		7		10		7	33			15.2
		97	First	260 mg/m^2^ q3w									42			
		132	Further	260 mg/m^2^ q3w									27			13.2
Blum JL 2007 [6]	Phase II Multicenter	106	Further	100 mg/m^2^ qw 3/4	49	18	62	19	25	8	37	5	14	26	3	9.2
		75	Further	125 mg/m^2^ qw 3/4	64	34	66	36	51	19	45	12	16	37	3.5	9.1
Guan ZZ 2009 [17]	Phase II RCT Multicenter	104	Further	260 mg/m^2^ q3w	69	42	64	24	76	7	17		54	71	7.6	17.8
Mirtsching B 2011 [29]	Phase II Multicenter	72	First	125 mg/m^2^ qw 3/4	14	11	11	1.4	54	8.3	58	6.9	42.2	68.8	14.5	29
Gradishar WJ 2009/2012 [15, 16]	Phase II RCT Multicenter	76	First	100 mg/m^2^ qw 3/4	80	25			58	9	34	0	63	83	7.5	22.2
		74	First	150 mg/m^2^ qw 3/4	92	44			68	22	47	4	74	91	14.6	33.8
		76	First	300 mg/m^2^ q3w	93	44			73	21	36	5	46	72	10.9	27.7
Brezden B 2016 [4]	Phase II Multicenter	47	First	100 mg/m^2^ qw 3/4	14.9	9			46.8	0	70		30	51	6	20.9
		76	First	100 mg/m^2^ qw 3/4	17.1	9			57.9	0	72		28	57	6.7	20
Ranade AA 2013 [36]	Phase II RCT Multicenter	55	Mixed	220 mg/m^2^ q3w		28				1.5			40	67		
		53	Mixed	300 mg/m^2^ q3w		38				17			40	72		
Palumbo R 2015 [35]	Phase II Multicenter	52	Further	260 mg/m^2^ q3w	77	21.2	88.5	25	48.1	5.8	27	0	48.1	76.9	8.9	
Fabi A 2015 [14]	Phase II Single center	42	Further	Mixed	95	70	95	25	80	12	72	13	23.8	50	4.6	
		32	Further	125 mg/m^2^ qw 3/4									28.1	50		
		10	Further	260 mg/m^2^ q3w									10	50		
Hurria A 2015 [21]	Phase II Multicenter	39	Mixed	100 mg/m^2^ qw 3/4	26	3	36	5	8	0	31	5	31	69	5.7	19.4
Andres FT 2015 [1]	Phase II RCT Multicenter	21	Mixed	100 mg/m^2^ qw 3/4	38	14			48	5	54	10	38	52	3.7	
Jain MM 2016 [25]	Phase III RCT Multicenter	58	Mixed	260 mg/m^2^ q3w	33	21	28	16	60	17	36	7	43	75	7.9	
Yamamoto S 2017 [42]	Phase II Multicenter	35	Mixed	180 mg/m^2^ q3w		46				0			23	51	6.5	44.7
Tamura K 2017 [39]	Phase II RCT Multicenter	98	First	150 mg/m^2^ qw 3/4	97	78	96	58	88	22	33	1	61.2	96.9	11.2	42.4
Bernardo A 2017 [5]	Cohort	209	Further	Mixed	9.4	3			29.9	2.1	27.4	1.7	32.1	57.7		18
		68	Further	125 mg/m^2^ qw 3/4	8.7	1.1			17.4	2.2	12	1.1	31.5	54.3		16.9
		121	Further	260 mg/m^2^ q3w	9.9	4.2			38	2.1	37.3	2.1	32.4	59.9		18
Gennari A 2018 [18]	Phase II RCT Multicenter	83	First	150 mg/m^2^ q2w	55.4	21.7			69.5	8		48.2	47	65.1	7.9	25.8
		86	First	100 mg/m^2^ qw 3/4	68.6	27.9			73.2	5.8		46.5	54.7	68.6	9	26.2
		86	First	75 mg/m^2^ qw	72.2	24.4			70	5.8		10.5	44.7	60.5	8.5	25.5
Marschner N 2018 [32]	Cohort Multicenter	697	Mixed	Mixed		4		7.5	39.6	4.3	20.8	1.3	37.2	68.3	5.9	15.6
		491	Mixed	≤150 mg/m^2^ qw									39.1	68.8	6	16.3
		194	Mixed	220-260 mg/m^2^ q3w									33	67.5	5.7	15.1
Hurria A 2019 [23]	Phase II Multicenter	40	Mixed	100 mg/m^2^ qw 3/4	44	11	18	3	10	5	55	5	35	75	6.5	21.2
Ciruelos E 2019 [7]	Phase II RCT Multicenter	16	First	100 mg/m^2^ qw 3/4	37.5	0	50.1	6.3	81.3	0	43.9	6.3	37.5			
		14	First	150 mg/m^2^ q2w	18.8	0	25.1	6.3	62.6	0	87.6	0	12.5			
		16	First	150 mg/m^2^ qw 3/4	64.2	50	50	28.6	78.6	35.7	64.3	14.3	42.9			
Hara F 2019 [22]	Phase II RCT Multicenter	48	Mixed	180 mg/m^2^ q3w	50	14.6	60.4	14.6	81.3	8.3	70.8	0	37.8		6.8	
		45	Mixed	220 mg/m^2^ q3w	73.3	37.7	77.8	26.6	84.4	8.9	77.8	0	44.1		7.3	
		47	Mixed	260 mg/m^2^ q3w	57.4	25.4	66	19.1	91.5	31.9	80.9	2.1	48.7		6.7	
Schmid P 2020 [38]	Phase III RCT Multicenter	449	First	100 mg/m^2^ qw 3/4	15	8.2			23	2.7	44	3	45.9	72.4	5.5	18.7

### Nab-paclitaxel treatment patterns

In total, 3287 MBC patients treated with nab-paclitaxel monotherapy were included in the current study. In our analysis, 1685 (51.26%) MBC patients received nab-paclitaxel as first-line therapy, 640 patients (19.47%) as further-line therapy, and the remaining 962 MBC patients (29.27%) as mixed (first or further)-line therapy. Furthermore, a majority of the MBC patients (*n* = 1966, 60.40%) had nab-paclitaxel administered weekly, 1190 MBC patients (36.56%) had nab-paclitaxel administered triweekly, and 99 MBC patients (3.04%) had biweekly administration of 150 mg/m^2^ nab-paclitaxel. Among 1190 MBC patients with a triweekly nab-paclitaxel schedule (q3w), 194 patients (16.30%) were administered an imprecisely reported dosage of 220–260 mg/m^2^, 192 patients (16.13%) were administered a dosage of 300 mg/m^2^, 621 patients (52.18%) were administered 260 mg/m^2^, 100 patients (8.40%) were administered 220 mg/m^2^, and 83 patients (6.97%) were administered 180 mg/m^2^. Among the 1966 MBC patients with a weekly nab-paclitaxel schedule, 956 patients (48.62%) received a dosage of 100 mg/m^2^ qw 3/4, 247 patients (12.56%) received 125 mg/m^2^ qw 3/4, 186 patients (9.5%) received 150 mg/m^2^ qw 3/4, and 86 patients (4.4%) received at 75 mg/m^2^ qw. Additionally, 491 MBC patients (24.97%) who received weekly nab-paclitaxel monotherapy in the NABUCCO study were reported to have had a ≤ 150 mg/m^2^ qw schedule.

### Nab-paclitaxel monotherapy safety profiles

All 3287 MBC patients were included in the safety analysis. Neutropenia, leukopenia, peripheral sensory neuropathy and fatigue were the four chosen representative TRAEs of nab-paclitaxel monotherapy.

**Fig. 2:**
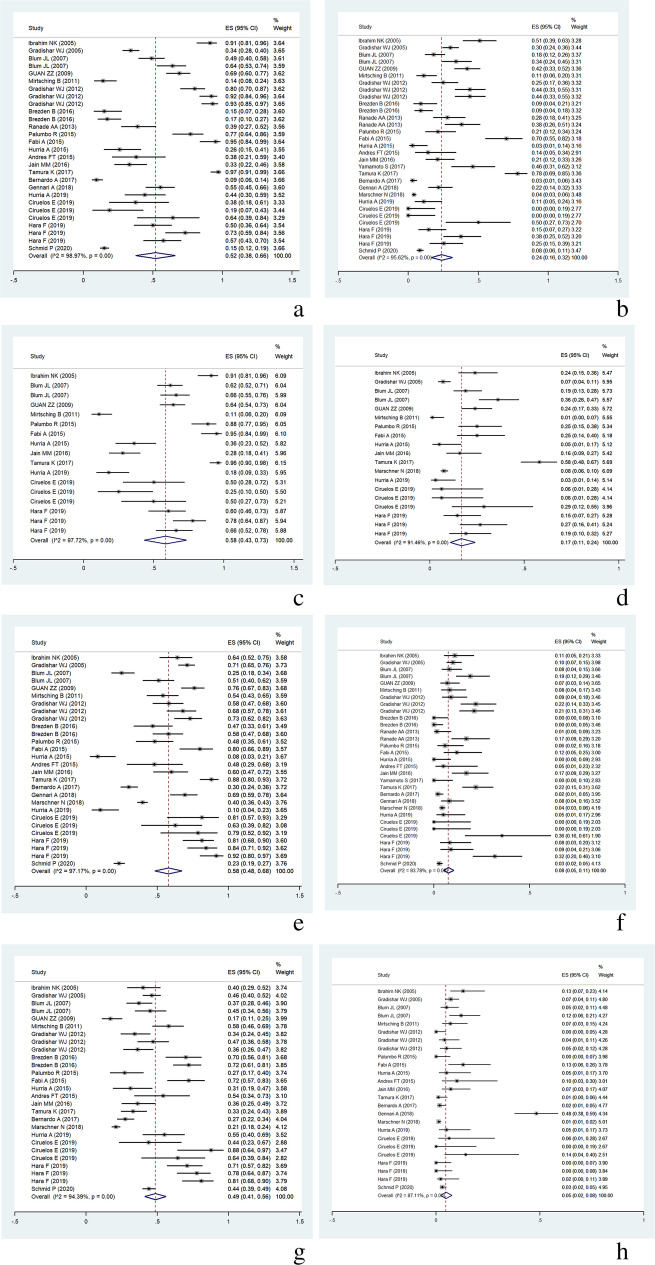
**a** Incidence of neutropenia, all grades. **b** Incidence of grade 3/4 neutropenia. **c** Incidence of leukopenia, all grades. **d** Incidence of grade 3/4 leukopenia. **e** Incidence of peripheral sensory neuropathy, all grades. **f** Incidence of grade 3/4 peripheral sensory neuropathy. **g** Incidence of fatigue, all grades. **h** Incidence of grade 3/4 fatigue

According to our study, 22 included studies with 31 individual groups reported the incidence of treatment-related neutropenia after nab-paclitaxel monotherapy in 3287 MBC patients. After data integration, the overall incidence of all-grade neutropenia was 52% (95% CI, 38–66%), and the incidence of grade 3/4 neutropenia was 24% (95% CI, 16–32%) (Fig. 2a, b). Nineteen individual groups reported the incidence of chemotherapy-related leukopenia, with an overall incidence of all-grade leukopenia of 58% (95% CI, 43–73%), and an incidence of grade 3/4 leukopenia of 17% (95% CI, 11–24%) (Fig. 2c, d). Overall, the incidence of all-grade and grade 3/4 peripheral sensory neuropathy were reported for 3287 MBC patients in 31 individual groups. The overall incidence of all-grade peripheral sensory neuropathy was 58% (95% CI, 48–68%), and the incidence of grade 3/4 peripheral sensory neuropathy was only 8% (95% CI, 5–10%) (Fig. 2e, f). Twenty-seven individual groups reported the incidence of treatment-related fatigue. The overall incidence of all grades of fatigue was 49% (95% CI, 41–56%), and the incidence of grade 3/4 fatigue was 5% (95% CI, 2–8%) following nab-paclitaxel administration (Fig. 2g, h).

### Risk factors affecting TRAEs

**Fig. 3:**
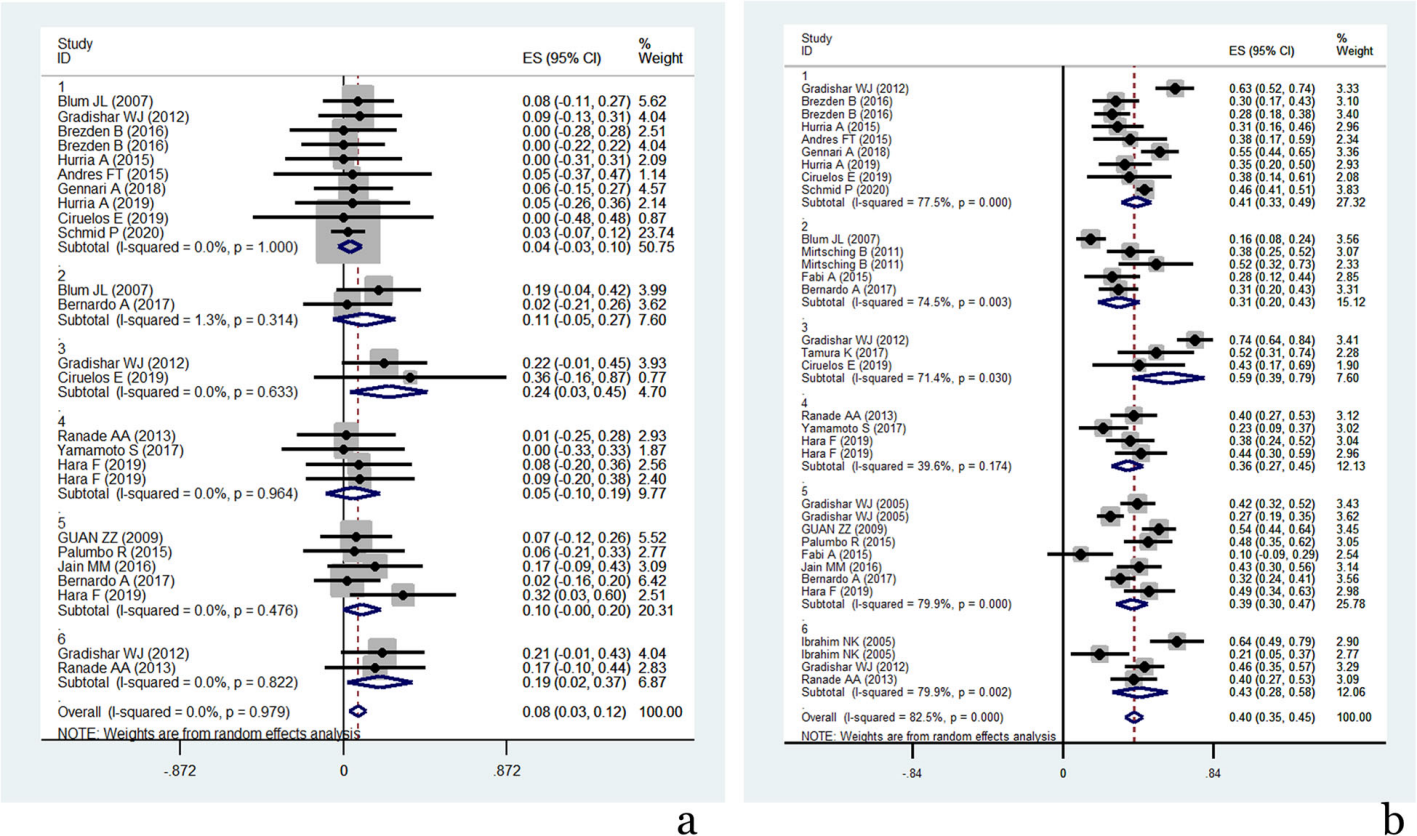
**a** Incidence of grade 3/4 peripheral sensory neuropathy related to different nab-paclitaxel dosages. **b** Overall response rate related to different nab-paclitaxel dosages (1=100mg/m2; 2=125mg/m2; 3=150mg/m2; 4=<260mg/m2; 5=260mg/m2; 6=300mg/m2. nab-paclitaxel dosage.)

According to the results of the meta-regression analysis, treatment lines, nab-paclitaxel dosage, and nab-paclitaxel schedule did not affect the incidence of all-grade neutropenia or grade 3/4 neutropenia. However, grade 3/4 neutropenia occurred less frequently in Her-2-negative patients than in the entire population (Coef value = 0.063, *P* = 0.046). All-grade leukopenia seemed to occur more frequently in MBC patients treated with nab-paclitaxel as further-line therapy (Coef value = 0.366, *P* = 0.056). Treatment lines, patient population, and the schedule of nab-paclitaxel administration did not contribute to the development of all grades or grade 3/4 peripheral sensory neuropathy. However, the dosage of nab-paclitaxel monotherapy seemed to be a potential independent risk factor affecting the incidence of grade 3/4 peripheral sensory neuropathy (Coef value = 0.201, *P* = 0.078), as the *P* value almost reached 0.05. Meanwhile, an obvious clinical trend showed that grade 3/4 peripheral sensory neuropathy was more frequently recorded in patients with higher nab-paclitaxel dosages (Fig. 3a). Nab-paclitaxel-related fatigue (all grades) occurred more frequently in MBC patients receiving further-line monotherapy (Coef value = − 0.239, *P* = 0.032).

### Nab-paclitaxel monotherapy efficacy outcomes

All 3287 MBC patients were included in the efficacy analysis. ORR, CBR, PFS, and OS were the chosen efficacy endpoint indices of nab-paclitaxel monotherapy.

Thirty-three individual groups reported the ORR as the major efficacy outcome for nab-paclitaxel monotherapy. After data integration, the cumulative ORR was 40% (95% CI, 35–45%). Twenty-five individual groups reported the CBR, with the cumulative estimate being 66% (95% CI, 59–73%). Additionally, complete remission (CR) was noted in 23 individual groups, and the cumulative ratio of CR was only 3% (95% CI, 2–5%). Partial remission (PR) and stable disease (SD) were more common than CR, with cumulative proportions reaching 38% (95% CI, 32–44%) and 28% (95% CI, 24–31%), respectively.

Twenty-three individual groups with 2399 MBC patients reported PFS as an outcome after nab-paclitaxel monotherapy. The median PFS ranged from 3.7 to 14.6 months, and the overall median PFS was 7.64 months (95% CI, 6.89–8.40 months). OS was reported in 14 studies with 17 individual groups containing 2472 MBC patients. The median OS ranged from 15.2 to 44.7 months, and the overall median OS was 24.51 months (95% CI, 21.25–27.78 months).

**Fig. 4:**
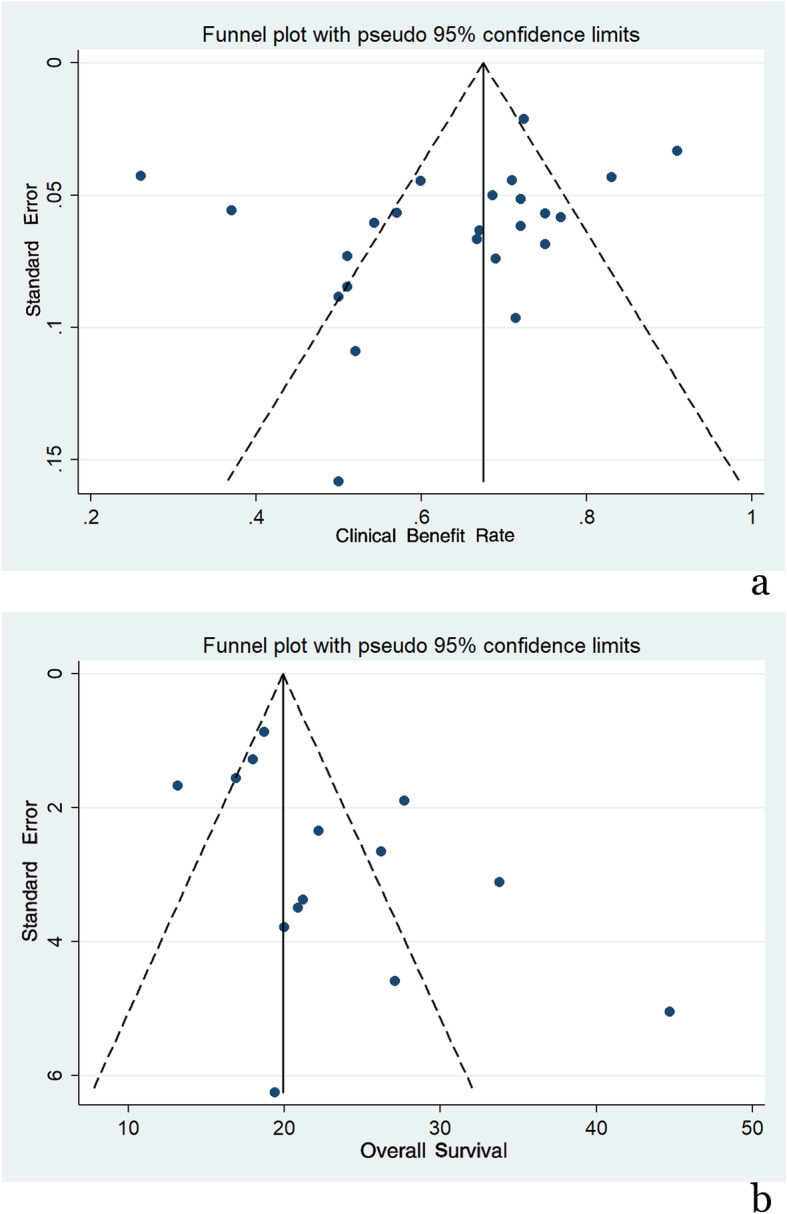
**a** Funnel plot of CBR. **b** Funnel plot of OS

Begg’s and Egger’s tests revealed publication bias with respect to CBR and OS. The corresponding funnel plots are presented in Fig. 4a, b.

### Risk factors affecting efficacy outcomes

According to the meta-regression analysis, we found that patients treated with nab-paclitaxel monotherapy in further-line therapy suffered from an unfavourably lower ORR (Coef value = − 0.18, *P* = 0.006) relative to patients treated with nab-paclitaxel monotherapy at other points in their treatment. In the subsequent subgroup analysis, the ORR was 48.2% (95% CI, 41–45%) in patients treated with nab-paclitaxel in first-line therapy, 40.1% (95% CI, 35.1–45%) in the mixed line (first or further line) treatment with nab-paclitaxel, and 27.2% (95% CI, 20.1–34.3%) in nab-paclitaxel treatment in further-line treatment. Similar results and statistical significance were also obtained relative to CBR (Coef value = − 0.176, *P* = 0.037), with CBR estimated at 55.3% (95% CI, 43.2–67.4%) with nab-paclitaxel in further-line treatment, 68.7% (95% CI, 62.5–75%) with nab-paclitaxel in first-line treatment, and 68.8% (95% CI, 63.5–74%) with nab-paclitaxel in mixed-line treatment. The schedule of nab-paclitaxel administration (weekly, biweekly or triweekly) did not affect the ORR (Coef value = − 0.212, *P* = 0.116). However, within the same schedule of nab-paclitaxel administration, patients appeared to have a superior ORR with increasing doses of nab-paclitaxel (Coef value = 0.081, *P* = 0.044) (Fig. 3b).

Patients who received first-line nab-paclitaxel monotherapy were demonstrated to have a longer median PFS than those who received nab-paclitaxel monotherapy during mixed-line therapy. The median PFS was 8.01 months (95% CI, 6.83–9.18 months) in the first-line group and 6.55 months (95% CI, 5.69–7.4 months) in the mixed-line group. Patients who received further-line nab-paclitaxel monotherapy were demonstrated to have a shorter median OS than those who received first- and mixed-line nab-paclitaxel monotherapy therapy; the median OS values were 16.18 months (95% CI, 13.41–18.94 months), 24.41 months (95% CI, 20.4–28.42 months), and 28.44 months (95% CI, 12.75–44.13 months) after further-, first-, and mixed-line therapy, respectively. Similarly, similar to the ORR, the schedule of nab-paclitaxel administration (weekly, biweekly or triweekly) did not affect the median PFS (Coef value = − 2.77, *P* = 0.162) or median OS (Coef value = 6.27, *P* = 0.623). The median PFS was 6.94 months (95% CI, 5.92–7.96 months) for weekly therapy and 7.71 months (95% CI, 6.90–8.52 months) for q3w nab-paclitaxel monotherapy. However, within the same schedule of nab-paclitaxel administration, patients appeared to have a longer PFS along with an increasing dosage of nab-paclitaxel (Coef value = 2.68, *P* = 0.03). This association was not found with respect to the median OS (Coef value = 6.27, *P* = 0.62). Risk factors affecting nab-paclitaxel monotherapy-related adverse events and efficacy outcomes are presented in Table 3.

**Table 3:** Risk factors affecting nab-paclitaxel monotherapy related adverse events and efficacy outcomes by meta-regression analysis

Effect Index	Risk Factors	Coef. value	Std. Err.	T value	*P* value	95% Confidence Interval
Incidence of neutropenia 3/4 grade	Her-2 negative	−0.136	0.063	−2.14	0.046	−0.268 to −0.003
Incidence of leukopenia all grades	Treatment line	0.366	0.148	2.48	0.056	−0.014 to 0.746
Incidence of neuropathy 3/4 grade	Nab-paclitaxel dosage	0.201	0.107	1.87	0.078	−0.025 to 0.427
Incidence of fatigue all grades	Treatment line	−0.239	0.100	−2.40	0.032	−0.455 to − 0.024
ORR	Treatment line	−0.180	0.059	−3.03	0.006	−0.302 to − 0.058
	Nab-paclitaxel dosage	0.171	0.081	2.11	0.044	0.005 to 0.338
CBR	Treatment line	−0.176	0.077	−2.29	0.037	−0.340 to − 0.012
PFS	Treatment line	1.398	0.635	2.20	0.045	0.036 to 2.760
	Nab-paclitaxel dosage	2.683	1.114	2.41	0.030	0.295 to 5.071
OS	Treatment line	−18.909	8.210	−2.30	0.040	−36.797 to −1.021

## Discussion

Taxanes are regarded as the most widely used and effective single antitumour agent in the treatment of MBC [3]. Nab-paclitaxel, a relatively younger member of the taxane family, has gained increasing favour in treating MBC due to its special antitumour characteristics and low toxicities, as demonstrated over the past 15 years. However, nab-paclitaxel was not frequently administered in patients with breast cancer in China mainly because of its high cost (approximately 6200 dollars for four cycles). Moreover, nab-paclitaxel could only be purchased personally, as the expense was beyond the range of routine healthcare coverage. Since Jan. 2020, nab-paclitaxel has been listed as one of the drugs with centralized procurement in large quantities; therefore, it has become more affordable to patients (approximately 1700 dollars for four cycles); the same nab-paclitaxel is now available at quite a different price. We were inspired by this significantly different cost and wondered what exactly nab-paclitaxel monotherapy could provide for MBC patients in terms of both TRAEs and clinical benefits. Although a proportion of studies reported that nab-paclitaxel monotherapy had acceptable safety profiles, higher disease control rates and improved survival in the management of MBC [2, 16, 24, 34], no systematic research has discussed this issue with pooled integration of the data.

In our study, the safety profiles of nab-paclitaxel mono-treatment were first analysed. Neutropenia and leukopenia were the most common haematologic adverse events (AEs) of nab-paclitaxel, and some authors even reported that the incidence of grade 3/4 neutropenia after nab-paclitaxel monotherapy was higher than 50% [37]. According to our analysis, after integrating each individual group, the overall incidence of all-grade neutropenia and leukopenia was 52 and 58%, and the incidence of grade 3/4 neutropenia and leukopenia was 24 and 17%, respectively. Furthermore, across the majority of studies, these haematologic adverse events were generally considered uncomplicated and could be rapidly resolved after treatment interruption, dose reduction and granulocyte colony-stimulating factor (G-CSF) supplementation. Although neutropenia and leukopenia are known to be dose-limiting, we found that the incidence of all-grade and grade 3/4 neutropenia and leukopenia were not correlated with the nab-paclitaxel dosage or schedule, which indicated that neutropenia or leukopenia might not be a dose- or schedule-dependent adverse event. We still found that Her-2 expression status seemed to be correlated with the incidence of grade 3/4 neutropenia, as the incidence of grade 3/4 neutropenia was significantly lower in the Her-2-negative population. Moreover, the differences in patient populations (Her-2-negative or triple negative) among studies might be a potential source of heterogeneity leading to different incidences of grade 3/4 neutropenia. However, the actual reason remains unknown.

Peripheral sensory neuropathy is a common and specific adverse event of taxane-based therapy. In our analysis, following nab-paclitaxel monotherapy, the overall incidence of all-grade peripheral sensory neuropathy was 58%, and the overall incidence of grade 3/4 neuropathy was 8%. Interestingly, unlike haematologic AEs, we found that the nab-paclitaxel dosage seemed to be a potential risk factor affecting the incidence of grade 3/4 peripheral sensory neuropathy, and this was another potential source of heterogeneity of the included studies. Although statistical significance was not observed (*P* = 0.078), a relatively obvious trend could be noted: grade 3/4 peripheral sensory neuropathy was more frequently recorded in patients with higher nab-paclitaxel dosages. Furthermore, in those who received fixed weekly or triweekly nab-paclitaxel monotherapy, the incidence of grade 3/4 peripheral sensory neuropathy increased if the nab-paclitaxel dosage increased. This was in accordance with the results reported by Ciruelos et al. [7], who found that grade 3 peripheral neuropathy, which is known to be cumulative, was deemed to be taxane-related.

Concerning the efficacy outcomes, our analysis showed that nab-paclitaxel monotherapy could provide a 40% ORR, a 60% CBR, a median PFS of 7.64 months and a median OS of 24.51 months for the overall population of patients with MBC who received various doses, schedules, and regimens of nab-paclitaxel across all lines of therapy. Nevertheless, in MBC patients who received nab-paclitaxel monotherapy as the first-line treatment, the nab-paclitaxel efficacy outcomes were more encouraging, with a 48.2% ORR, a 68.7% CBR, a median PFS of 8.01 months and a median OS of 24.41 months. These results are superior to those of a previous real-life study with a sizeable sample, which showed a median time to next therapy or death (TNTD) of 6.1 months and a median OS of 17.4 months in patients receiving nab-paclitaxel monotherapy for ≥ first-line treatment of MBC [26]. In the current study, we suggested that treatment line and nab-paclitaxel dosage were risk factors affecting ORR and median PFS. Patients treated with nab-paclitaxel monotherapy in further- or mixed-line therapy endured lower ORR and shorter median PFS compared with patients treated with nab-paclitaxel monotherapy in first line. A better ORR or longer PFS appeared to be related to the dosage increase of nab-paclitaxel. Further study is needed to confirm the relationship between dosage and therapeutic effect because of the heterogeneity existed at present.

Nab-paclitaxel can be as administered on a triweekly schedule, but it is also justifiable to administer it in various weekly schedules in patients with MBC. It is particularly true that different opinions exist among the experts regarding the optimal schedule for nab-paclitaxel administration [8]. Findings from a randomized study by Gradishar [15] suggested that nab-paclitaxel administered weekly for 3 weeks at a dose of 150 mg/m^2^ followed by a 1-week break is more effective in terms of PFS than nab-paclitaxel administered at 100 mg/m^2^ weekly. In our study, we found that a weekly administration schedule was more frequently used than a triweekly schedule. However, according to our analysis, regardless of which nab-paclitaxel schedule (weekly, biweekly or triweekly) was chosen, the nab-paclitaxel efficacy outcomes were not affected. The recently published NABUCCO study also showed no differences in terms of the clinical activity of nab-paclitaxel according to the schedule used [32]. This finding is of particular interest for clinical practice. As the schedule of nab-paclitaxel administration is not proven to be correlated with the efficacy outcomes, we suggested that nab-paclitaxel can be safely used with weekly and triweekly schedules, leaving the choice to the physician according to the patient’s needs and preference, with a careful balance between activity and potential toxicity. In other words, nab-paclitaxel offers flexible scheduling. Moreover, the treatment line, rather than the nab-paclitaxel dosage, was demonstrated to be the only risk factor affecting median OS. Indeed, the assessment of OS was considered to be more objective than the assessment of PFS. These findings might partially guide us in terms of how nab-paclitaxel could be used in clinical practice on the basis of the current data; that is, for patients with higher tumour burden (visceral metastatic disease or > 2 metastatic lesions) who need immediate disease control could receive a maximum-tolerated dosage of nab-paclitaxel, such as 300 mg/m^2^ q3w or 150 mg/m^2^ qw 3/4. These regimens are likely to bring patients a better ORR and longer PFS, but the overall survival might not be changed.

In this systematic review and meta-analysis, we demonstrated that nab-paclitaxel mono-chemotherapy was a low-toxicity and effective strategy in the palliative management of MBC patients. Both weekly and triweekly nab-paclitaxel mono-chemotherapy seemed to be effective for MBC with generally reasonable toxicity profiles. A higher ORR and longer PFS and OS were observed in patients treated with nab-paclitaxel as first-line therapy. Increasing nab-paclitaxel dosage was more likely to result in better tumour control (higher ORR and PFS); however, changing the nab-paclitaxel schedule might have no benefit on ameliorating the survival outcomes.

A few limitations of our research need to be mentioned in particular. Firstly, despite the included studies were designed prospectively, some publications were not of high quality. Secondly, the strength of part of the conclusions we came to were limited due to the significant heterogeneity encountered, the publication bias and the lack of statistical difference. Thirdly, the Her-2-negative population, nab-paclitaxel dosage and treatment line were demonstrated to be potential sources of heterogeneity among studies; however, other sources of heterogeneity still existed. Forthly, several characteristics of the MBC patients, such as race, menopausal status, and different metastatic sites, were not extracted or analysed, which might lead to uncomprehensive results.

## Conclusions

The benefits brought by nab-paclitaxel mono-chemotherapy in the treatment of MBC are considerable while the harm is generally manageable. What is more, there are some points that need to be made. First of all, both weekly and triweekly nab-paclitaxel mono-chemotherapy tend to be effective for MBC, but changing the nab-paclitaxel schedule may have no benefit in terms of survival outcomes. And then, a higher ORR and longer PFS and OS can be probably achieved in patients treated with nab-paclitaxel as the first-line therapy. Moreover, increasing nab-paclitaxel dosage seems to result in better tumour control (higher ORR and PFS). Last but not least, in consideration of the substantial heterogeneity among included studies, further study and validation are needed to enhance the accuracy of the conclusions we got.

## Supplementary Information

**Additional file 1: Supplement 1.** Quality assessment of the included studies for MBC.

## Data Availability

All data generated or analysed during this study are included in the published articles.

## Abbreviations

MBC: Metastatic breast cancer
ORR: Overall response rate
CBR: Clinical benefit rate
TTP: Time to progression
OS: Overall survival
TRAEs: Treatment-related adverse events
PFS: Progression free survival
HER-2: Human epidermal growth factor receptor-2
RCTs: Randomized controlled trials
FDA: Food and Drug Administration
QOL: Quality of life
PRISMA: Preferred Reporting Items for Systematic Reviews and Meta-Analyses
ASCO: American Society of Clinical Oncology
ESMO: European Society of Medical Oncology
SABCS: San Antonio Breast Cancer Symposium
MINORS: Methodological Index for Non-randomized Studies
CI: Confidence interval
CR: Complete remission
PR: Partial remission
SD: Stable disease
AEs: Adverse events
G-CSF: Granulocyte colony-stimulating factor
TNTD: Time to next therapy or death
ECOG: Eastern Cooperative Oncology Group
