# Supplementary material for: A systematic review and meta-analysis of nab-paclitaxel mono-chemotherapy for metastatic breast cancer
Source: BMC Cancer. 2021 Jul 18;21:830. doi: 10.1186/s12885-021-08441-z (PMC8286600; doi:10.1186/s12885-021-08441-z)
Supplement: Supplementary file 1 — Additional file 1: Supplement 1. Quality assessment of the included studies for MBC. [file 12885_2021_8441_MOESM1_ESM.docx]

Supplement 1 Quality assessment of the included studies for MBC

| **Author** | **Year** | **Cochrane** | **NOS** | **MINORS**  **non-control** | **MINORS**  **control** |
| --- | --- | --- | --- | --- | --- |
| **Gradishar WJ** | 2005 | Grade A |  |  |  |
| **GUAN ZZ** | 2009 | Grade A |  |  |  |
| **Gradishar WJ** | 2012 | Grade A |  |  |  |
| **Ranade AA** | 2013 | Grade A |  |  |  |
| **Andres FT** | 2015 | Grade A |  |  |  |
| **Jain MM** | 2016 | Grade A |  |  |  |
| **Tamura K** | 2017 | Grade A |  |  |  |
| **Gennari A** | 2018 | Grade A |  |  |  |
| **Ciruelos E** | 2019 | Grade A |  |  |  |
| **Hara F** | 2019 | Grade A |  |  |  |
| **Schmid P** | 2019 | Grade A |  |  |  |
| **Brezden B** | 2013 |  | 9 |  |  |
| **Ibrahim NK** | 2005 |  |  | 14 |  |
| **Mirtsching B** | 2011 |  |  | 16 |  |
| **Fabi A** | 2015 |  |  | 16 |  |
| **Hurria A** | 2015 |  |  | 16 |  |
| **Palumbo R** | 2015 |  |  | 16 |  |
| **Yamamoto S** | 2017 |  |  | 15 |  |
| **Hurria A** | 2019 |  |  | 14 |  |
| **Blum JL** | 2007 |  |  |  | 22 |
| **Bernardo A** | 2017 |  |  |  | 21 |
| **Marschner N** | 2018 |  |  |  | 22 |
